# Tenderization effect of whelk meat using ultrasonic treatment

**DOI:** 10.1002/fsn3.686

**Published:** 2018-08-22

**Authors:** Jiamiao Hu, Shenghan Ge, Chenying Huang, Peter C. K. Cheung, Luan Lin, Yi Zhang, Baodong Zheng, Shaoling Lin, Xiujuan Huang

**Affiliations:** ^1^ Fujian Province Key Laboratory for the Development of Bioactive Material from Marine Algae College of Oceanology and Food Science Quanzhou Normal University Quanzhou China; ^2^ Faculty of Health Sciences University of Macau Taipa Macau SAR; ^3^ College of Food Science Fujian Agriculture and Forestry University Fuzhou China; ^4^ School of Life Sciences The Chinese University of Hong Kong Shatin Hong Kong; ^5^ Fujian Saifu Food Inspection Co. Ltd. Fujian 350011, Fuzhou P.R. China

**Keywords:** *Buccinum undatum*, response surface methodology, tenderization, ultrasound

## Abstract

This study was conducted to assess the potential application of ultrasonic treatment to enhance the tenderness of whelk (*Buccinum undatum*) meat. The optimum ultrasonic conditions for the maximum tenderization effect were determined using response surface methodology by a three‐level factorial Box–Behnken design for the optimization of three variables. The optimum conditions for the three variables found were as follows: ultrasound power at 200 W, treatment time for 9.6 min, and temperature at 45°C. The resulted tenderization effect was comparable to traditional enzymatic methods. Furthermore, disruption of muscle microstructure was observed in the ultrasonic‐treated whelk meat by scanning electron microscopy, while evaluations on physicochemical properties indicated the ultrasonic treatment has no significant undesirable effects on the quality of whelk meat including pH, water‐holding capacity, and lipid oxidation. In conclusion, this study showed the feasibility of ultrasonic treatment as a promising tenderization method for whelk meat without detrimental effects on its quality.

## 1 INTRODUCTION

*Buccinum undatum*, also known as whelk or waved buccinum, is a large, edible marine gastropod, encased in a stout, yellowish‐brown shell with lighter and darker spiral areas, belonging to the family of Buccinidae (Wen & Laursen, 1994). Whelk (*B. undatum*) is widely consumed as seafood around the world, especially in Asia and Europe (Lee & Park, 2004). However, the “rubber‐like” texture of whelk meat is still a problem to be solved. Indeed, this tough mouthfeel is a main reason for its low acceptability in some markets (Sanchezbrambila et al., 2006). Therefore, a number of processing treatments to improve this textural problem have been investigated in order to increase the acceptance of whelk meat by the consumer. To date, exogenous proteases derived from plants, such as papain and bromelain have been reported to be effective agents used for whelk meat tenderization (Ha, Bekhit, Carne, & Hopkins, 2012). However, these enzymatic methods often adversely affect the appearance and quality of the whelk meat.

Research in the past few decades has shown the potential of ultrasonic treatment as a technique to enhance tenderness and sensory properties of meat and its by‐products (Jayasooriya, Bhandari, Torley, & D'Arcy, 2004; Jayasooriya, Torley, D'Arcy, & Bhandari, 2007). Previous researchers have reported that ultrasound processing can successfully tenderize porcine meat (Jørgensen, Christensen, & Ertbjerg, 2005), beef longissimus and pectoralis muscles (Got et al., 1999), chicken breast meat (Li, Kang, Zou, Xu, & Zhou, 2015), jumbo squid meat (Hu et al., 2014), and cobia sashimi (Chang & Wong, 2012). However, the application of ultrasonic treatment as an efficient technique for whelk meat tenderization has not been fully exploited. In this study, we investigated the optimum conditions of the ultrasonic treatment for whelk meat tenderization using RSM by a three‐level factorial Box–Behnken design to optimize three important parameters including ultrasonic power, treatment time, and temperature. The change in the muscle tissue structure of ultrasonic‐treated whelk meat was measured by scanning electron microscopy to elucidate the possible mechanisms involved. In addition, the effects of ultrasonic treatment on the quality of tenderized whelk meat in terms of pH, water‐holding capacity, and lipid oxidation were also evaluated.

## 2 MATERIALS AND METHODS

### 2.1 Materials

Whelk meats were provided by the Putian Huilong SEAFOOD Co. Ltd. (Fujian, China) and stored at −18°C till use. The frozen whelk meat was thawed at 0–4°C overnight and cut into chunks with approximate size of 2 × 2 × 2 cm^3^.

Papain and bromelain were purchased from Solvay Enzymes Inc. (Elkhart, IN, USA).

### 2.2 Ultrasonication treatment

#### 2.2.1 Single‐factor experiments

The whelk meat samples were loaded into the ultrasonic processor (KQ‐100VDB; Kunshan Ultrasound Instrument Co., Kunshan, China) and subjected to different ultrasonic treatments with selected power of 100–250 W for 2–16 min at 10–60°C. The frequency was set to 45 kHz. The paired adjacent sample without ultrasonic treatment was used as the control.

#### 2.2.2 Box–Behnken design (BBD) test

Based on the single‐factor experiments, the Design Expert software (Version 8.0.6; Stat‐Ease Inc., Minneapolis, MN, USA) was used to design and analyze response‐surface experiments, which was applied to estimate the effect of 3 independent variables (including power, X1; time, X2; and temperature, X3) on the treated/untreated ratios of shear force (Y). Three‐dimensional surface response plots were generated by changing two variables within the experimental range and holding the other variable constant at the central point/middle level. The fitness of the polynomial model equation to the responses was evaluated by the coefficient of R square, as well as by the lack of fit using the *F* test.

### 2.3 Exogenous protease treatment

The whelk meats were tumbled in either papain or bromelain solution under different conditions with concentration ranging from 4%–8% at a temperature of 30–70°C for 10–30 min. After tumbling, the samples were washed in cold water to eliminate excess protease from the surface and subjected to the determination of shear force. The enzyme solutions added to the whelk meats was fixed at 25% of meat weight (w/w).

### 2.4 Measurement of physical properties

#### 2.4.1 Shear force

To investigate the influence of ultrasonic treatment on the texture of whelk meat, shear force was determined using a WBS blade fitted to Texture Analyzer TA‐XT Plus (Stable 199 Micro Systems Ltd., Godalming, Surrey, UK). The test conditions were set as follows: pretest speed of 1.0 mm/s, test speed of 1.0 mm/s, posttest speed of 1.0 mm/s; deformation ratio of 30% and a rest period between two cycles of 5 s; trigger force of 5.0 g; and data acquisition rate of 200 pulses per second. The probe always returned to the trigger point before the second cycle. After the second cycle, the probe returned to its initial position. Each sample was compressed at two positions to obtain six readings for the subsequent data analysis, with the measurements made by placing the whelk meat lying perpendicular to the direction of plunger travel. The shear force was calculated by the User Guide software (version 1.0; Stable Micro Systems Ltd.), and the results were compared with untreated samples.

#### 2.4.2 Water‐holding capacities (WHC)

Water‐holding capacities was measured according to the method described previously (Rawdkuen, Jaimakreu, & Benjakul, 2013). Briefly, 20 g of minced whelk meat was mixed with 30 ml of 0.6 M NaCl and stirred with a glass rod in a centrifuge tube for 1 min. After being kept at 4°C for 15 min, the mixtures were stirred again and then centrifuged at 3,000 *g* for 10 min at 4°C. The supernatant was measured, and the WHC was expressed as a percentage calculated by the following equation:

(1) $$\begin{array}{cc} \text{WHC}(\%) & \;=\;\text{Volume}\;\text{of}\;\text{NaCl}\;\text{before}\;\text{centrifuge}\;\\ & \frac{\text{‐ Volume}\;\text{of}\;\text{NaCl}\;\text{after}\;\text{centrifuge}}{\text{Volume}\;\text{of}\;\text{NaCl}\;\text{before}\;\text{centrifuge}} \end{array}\times 100\%$$

#### 2.4.3 Cooking yield

According to the method reported previously (Rawdkuen et al., 2013), whelk meat (10 g) were steamed for 1 min and then cooled to room temperature. The cooked sample was surface‐dried with a filter paper and reweighed immediately. The cooking yield was determined by calculating the difference in weight of the raw and cooked meats as below:

(2) $$\text{Cooking}\;\text{yield}\;\;\left(\%\right)=\frac{\text{cooked}\;\text{weight}}{\text{raw}\;\text{weight}}\times 100\%$$

### 2.5 Measurement of chemical properties

#### 2.5.1 pH measurement

The pH of whelk meat sample was measured with a penetrating electrode (OSH 12‐00 Metron, Poland) fitted to a digital pH meter (CPC‐501 Elmetron, Poland). The electrode was driven into meat sample at the depth of about 1 cm. The probe was calibrated at the temperature of 4°C, and the measurements were carried out in triplicate at random points in the sample.

#### 2.5.2 Measurement of lipid oxidation

The extent of lipid oxidation of the whelk meat was assessed by the 2‐thiobarbituric acid (TBA) method described previosuly (Brenesselova et al., 2015). In brief, the whelk meat sample (10 g) was mixed with 40 ml distilled water and homogenized for 1 min. After that 40 ml 5% trichloroacetic acid was added and the mixture was kept at room temperature for 10 min. After filtration, 5 ml of the aliqout was mixed with 5 ml thiobarbituric acid in a stopper fitted flask and incubated in hot water (95°C) for 45 min. After cooling, the amount of thiobarbituric acid‐reactive substances (TBARS values) formed was measured spectrophotometrically at an absorbance of 532 nm. TBARS values in the sample were obtained from a standard curve of malonaldehyde (MAD) and expressed as mg MAD/kg sample.

### 2.6 Microbial analysis

Samples (10 g of whelk meat) for microbiological analysis were homogenized by food processer (JYL‐C012; Joyoung Co., Ltd. China) in 90 ml sterile water. This initial sample suspension and its subsequent decimal dilutions were prepared in accordance with the procedures described previously (Penfornis & Pochampally, 2016) and all of them were inoculated on LB agar plates. After incubation at 37°C for 48 hr, the number of colony‐forming units (CFUs) was counted under a light microscope. Each assay was performed in triplicate.

### 2.7 Scanning electron microscopy (SEM)

The microstructure of whelk meat was obtained using a field emission scanning electron microscope (SEM) (Nova Nano SEM 230, FEI, Netherlands). The untreated and treated whelk meats were cut with a scalpel into pieces (1 × 1 × 1 cm^3^) and fixed with 3% glutaradehyde for 3 hr and then were rinsed for 1 hr with distilled water before being dehydrated with absolute ethanol. The dried samples were mounted on a bronze stub and sputter‐coated with gold (Eiko IB‐5; Hitachi, Tokyo, Japan). The microstructures of whelk meats were observed at three different magnifications (500×, 2,000× and 5,000×).

### 2.8 Sensory evaluation

The overall sensory quality of whelk meat samples was assessed by a sensory panel consisted of six trained members in our faculty. Briefly, the whelk meat samples were boiled in water for 10 min and then evaluated for its hardness, texture, color, and odor based on a 10‐point scale with a 2‐point score for five categories including excellent, good, fair/acceptable, poor, and very poor).

### 2.9 Statistical analysis

All experiments were performed at least three times. All data were presented as means ± SEM values. As for multiple group comparison, the significance of the differences among the treatment groups and their respective control groups were analyzed using GraphPad Prism 5.0 software (GraphPad Software Inc., San Diego, CA, USA). Statistical significance was assessed by either Student's *t* test or one‐way analysis of variance (ANOVA) followed by Tukey's multiple comparisons. Differences between means were considered statistically significant if *p *<* *0.05.

## 3 RESULTS

### 3.1 Selection of the parameters of ultrasonic treatment by single‐factor tests

**Figure 1:**
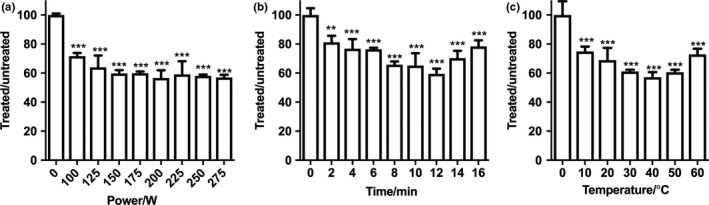
Effects of ultrasonic treatment with different ultrasound power, treatment time, and temperature on the shear force of whelk meat. Whelk meat was ultrasonic‐treated under various conditions ((a): power: 100–225 W; (b) treatment time 2–16 min, (c): temperature 10–60°C) and subsequently subjected for the shear force analysis. Data are presented as mean ± SEM. Statistical significance was assessed by one‐way ANOVA and Tukey's multiple comparison test with *^*^ *p*<0.01 and **^*^ *p*<0.001 when compared with untreated controls

According to previous reports, the tenderizing effects of ultrasonic treatment were mainly affected by factors including ultrasonic power, treatment time, and temperature. Thus, initial single‐factor tests were performed before the response surface experiments to screen the appropriate settings of the three variables including ultrasonic power, treatment time, and temperature for subsequent investigations. As shown in Figure 1, in the single‐factor test for the effect of ultrasonic power on meat tenderness, the treated/untreated ratio of the shear forces firstly decreased from 71.66 ± 1.24% to 56.60 ± 3.09% with increasing ultrasonic power from 100 to 200 W and then leveled off. Therefore, an ultrasonic power of 200 W was chosen as the central point/middle level for the subsequent experiments. The time range required for ultrasonic treatment was the second factor to be investigated. The maximum tenderization efficiency was peaked at 12 min, which was selected as a central point/middle level for response surface optimization. The effect of temperature on meat tenderness was then studied within the range of 10–60°C. The shear force decreased significantly (*p *< 0.05) with temperature increasing from 10 to 40°C and then increased slightly when the temperature rose from 40 to 60°C. Therefore, temperature range was set at 30–50°C for developing the response surface model. Although the above single‐factor tests revealed the possible ranges of the three variables/factors for ultrasonication treatment, this conventional method still might ignore the possible interaction effects arising between the three factors. Therefore, RSM was next employed to develop a model for optimization of tenderization of whelk meat.

### 3.2 Optimize ultrasonication treatment for whelk meat tenderization by RSM experiments

**Figure 2:**
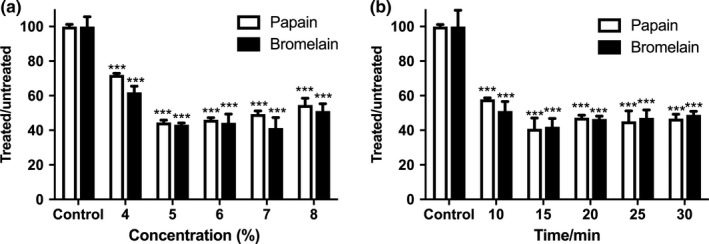
Effects of the exogenous proteases concentration and treatment time on the shear force of whelk meat. Whelk meat sample was tenderized with papain or bromelain under various conditions ((a): proteases concentration: 4%–8%; (b): treatment time: 10–30 min) and subsequently subjected for the shear force analysis. Data are presented as mean ± SEM. Statistical significance was assessed by one‐way ANOVA and Tukey's multiple comparison test with *^*^ *p*<0.01 and **^*^ *p*<0.001 when compared with untreated controls

To further optimize ultrasonic treatment parameters, the 3‐level factorial BBD for three variables with 17 runs was selected in this present study in order to optimize the conditions for the maximum tenderization of whelk meat. Based on the above single‐factor tests, we adopted an ultrasound power range of 180–220 W, a treatment time range of 9–15 min and a temperature range of 30–50°C for the three levels used in the subsequent RSM experiments (Table 1). The experimental response for 17 experimental runs are provided in Table 1, and Figure 2 presents the contour plots for the experimental response, whose regression equation can be expressed as the equation below (3):

(3) $$\begin{array}{cc} Y=\;\; & 917.75-5.56875X_{1}-24.98333X_{2}-6.7825X_{3}-0.033333X_{1}X_{2}\\ & +0.00375X_{1}X_{3}+0.05X_{2}X_{3}+0.013812X_{1}^{2}\\ & +1.19722X_{2}^{2}+0.07025X_{3}^{2} \end{array}$$

**Table 1:** Experimental conditions of whelk meat tenderization and responses for the 17 runs

Run	Independent variables			Response
	Power (W)	Time (min)	Temperature (°C)	Ratio of the shear forces (%) in treated/untreated samples
1	200 (0)	15 (+1)	50 (+1)	62
2	220 (+1)	12 (0)	30 (−1)	47
3	220 (+1)	9 (−1)	40 (0)	60
4	200 (0)	12 (0)	40 (0)	43
5	200 (0)	9 (−1)	30 (−1)	65
6	180 (−1)	9 (−1)	40 (0)	67
7	200 (0)	12 (0)	40 (0)	45
8	200 (0)	9 (−1)	50 (+1)	64
9	220 (+1)	15 (+1)	40 (0)	50
10	200 (0)	12 (0)	40 (0)	45
11	220 (+1)	12 (0)	50 (+1)	54
12	200 (0)	15 (+1)	30 (−1)	57
13	180 (−1)	15 (+1)	40 (0)	65
14	200 (0)	12 (0)	40 (0)	44
15	180 (−1)	12 (0)	50 (+1)	65
16	180 (−1)	12 (0)	30 (−1)	61
17	200 (0)	12 (0)	40 (0)	44

Analysis of variance (ANOVA) for this model is shown in Table 2. The determination coefficient (*R* ^2^ = 0.9837) indicated that the model was highly significant. The value of lack of fit test (*F*‐test) was higher than 0.05 (0.1197), which was not significant relative to the pure error, indicating that the fitting model was adequate to describe the experimental data. In an analysis of variance, *p*‐value and *F*‐value are used to reflect the significance of corresponding variables. As given in Table 2, comparing the linear, interaction, and quadratic terms, it can be found that ultrasonic power, treatment time, and temperature were highly significant to shear force with the *p*‐values lower than 0.01. Table 2 and the contour plots in Figure 2 also demonstrate the interaction effects between ultrasonic power with treatment time (X_1_X_2_) and treatment time with temperature (X_2_X_3_) were significant (*p *<* *0.05). No significant interaction effects between ultrasonic power and temperature (X_1_X_3_) could be found (*p *>* *0.05).

**Table 2:** Analysis of variance of the experimental results of the Box–Behnken design

Variables	Sum of squares	DF	Mean square	*F* value	*p*‐Value
Model	1,303.92	9	144.88	96.13	<0.0001
A‐Power	276.12	1	276.12	183.21	<0.0001
B‐Time	60.50	1	60.50	40.14	0.0004
C‐Temp	28.13	1	28.13	18.66	0.0035
AB	16.00	1	16.00	10.62	0.0139
AC	2.25	1	2.25	1.49	0.2613
BC	9.00	1	9.00	5.97	0.0445
A^2^	128.53	1	128.53	85.28	<0.0001
B^2^	488.84	1	488.84	324.35	<0.0001
C^2^	207.79	1	207.79	137.87	<0.0001
Residual	10.55	7	1.51		
Lack of fit	7.75	3	2.58	3.69	0.1197
Corrected total	1,314.47	16			
*R*‐squared	0.9920				
Adjusted *R*‐squared	0.9817				
Predicted *R*‐squared	0.9023				
C.V. %	2.22				

Based on the above results, a verification was performed to evaluate the optimal processing condition on the shear force. The optimal conditions were predicted to be at ultrasonic power of 203.27 W, treatment time of 9.61 min, and temperature of 45.21°C, under which the anticipated decrease in the treated/untreated ratio of shear force was 54.95%. Considering the practical operation of the ultrasonic equipment, the optimal conditions were set at an ultrasound power (X1) of 200 W, treatment time (X2) for 9.6 min, and temperature (X3) at 45°C, under which the experimental decrease in shear force was 55.17%. Such small discrepancy in the predicted and experimental value of the shear force suggested that the RSM model used was satisfactory and accurate.

**Figure 3:**
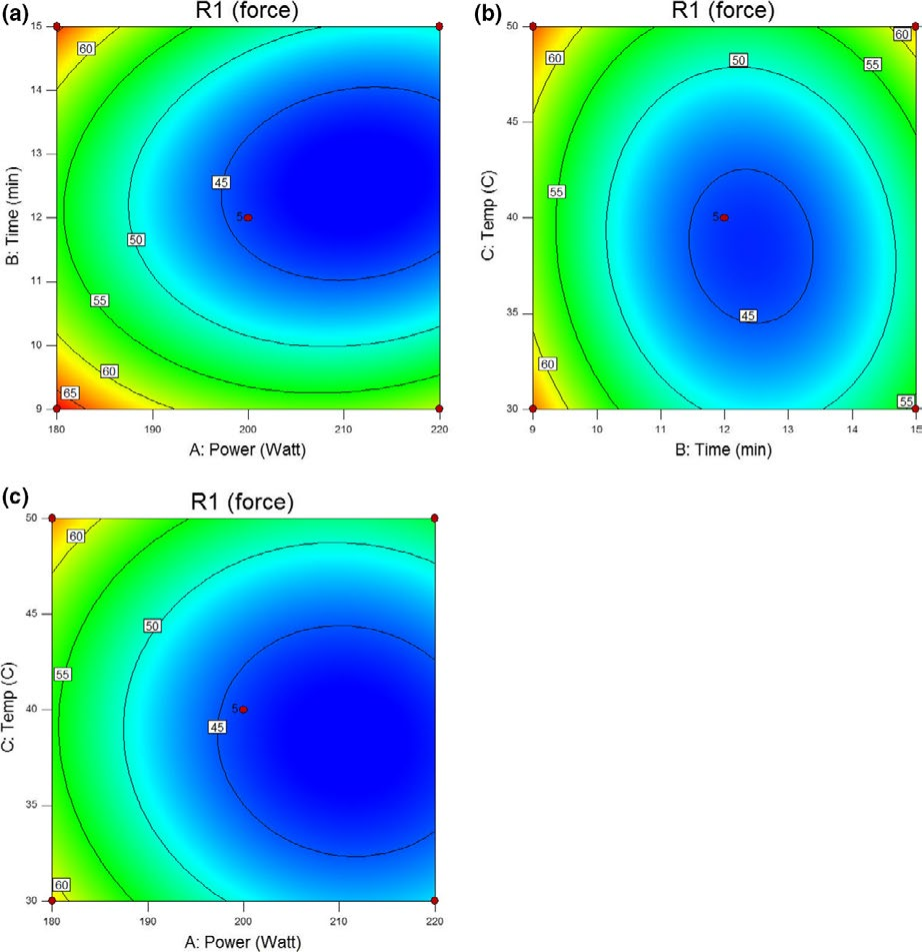
Two‐dimensional (2‐D) contour plots for the shear force of whelk meat subjected to the ultrasonic treatment with three variables (X1: ultrasound power, X2: treatment time, and X3: temperature). Contour plots showing the effect of (a): ultrasound power and treatment time; (b): ultrasound power and temperature; (c): treatment time and temperature on the shear force of whelk meat

In addition, the tenderization effects of ultrasonic treatment were also compared with traditional meat tenderizers including papain and bromelain. As shown in Figure 3, in the range of 0–8% (w/v), papain and bromelain reduced the shear force of the whelk meat by 44.52% and 41.27%, respectively, which was similar to the tenderization effects of ultrasonic treatment at the optimal condition, suggesting ultrasonic treatment can reach comparable tenderization effects compared to traditional exogenous protease tenderizers.

### 3.3 Effects of tenderization methods on the microstructure of whelk meats

**Figure 4:**
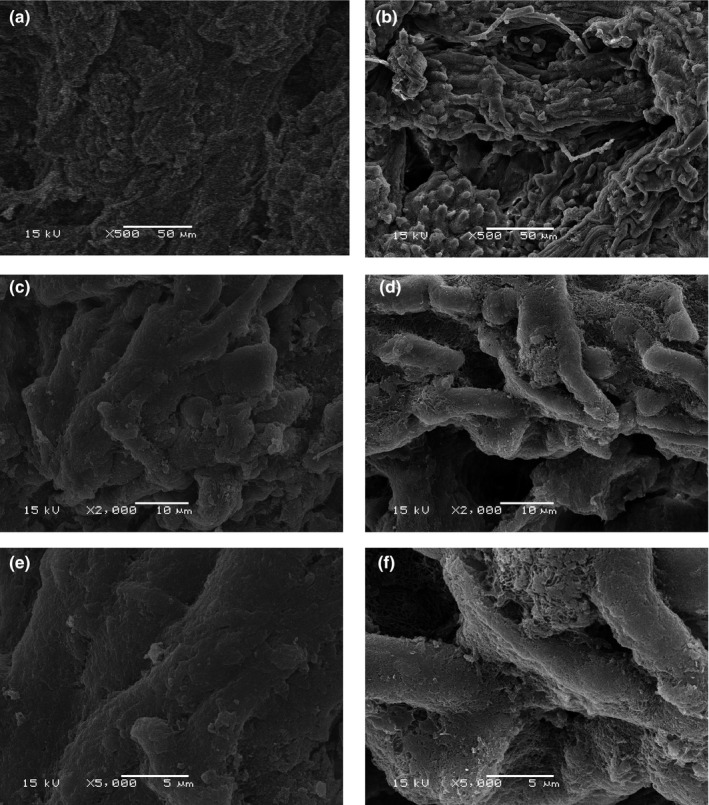
Scanning electron microscopy (SEM) photographs from control and ultrasonic‐treated whelk meat. (a, c, e) Whelk meat samples without ultrasonic treatment; (b, d, f) whelk meat samples subjected to ultrasonic treatment. The magnification was set as ×500 (a, b), ×2,000 (c, d) and ×5,000 (e, f)

As shown in Figure 4, scanning electron micrographs were applied to exam the microstructure of the untreated and sonicated whelk meats. The untreated whelk meat fiber microstructure was arranged very closely and tightly indicating muscle hardness (Figure 4a,c, and e). In contrast, ultrasonic‐treated whelk meat under the optimal processing conditions showed disruption fiber microstructure which were loosely packed, having more irregular appearance and larger cavities (Figure 4b,d, and f), indicating muscle softening.

### 3.4 Effects of optimized tenderization treatment on the quality parameters of whelk meat

**Figure 5:**
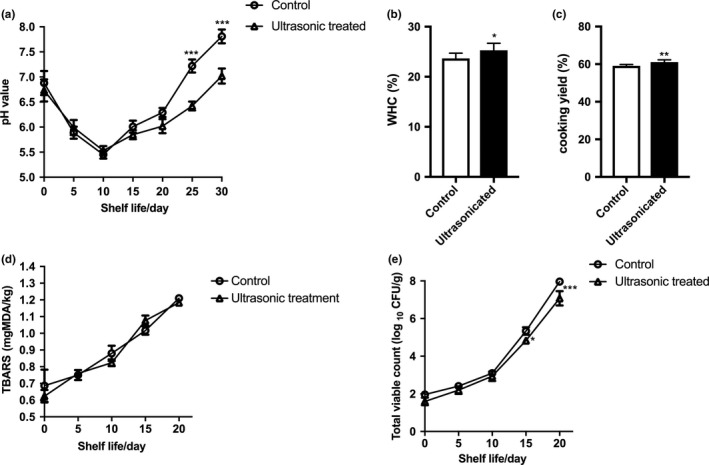
The effects of ultrasonic treatment on the physico‐chemical characteristics of whelk meat. Whelk meat sample was tenderized by ultrasonic treatment at RSM optimized condition. Changes of (a) pH, (b) water‐holding activity, (c) cooking yield, (d) TBARS, and (e) total viable count of ultrasonic‐treated samples were measured and compared with control samples without ultrasonic treatment. All samples were stored at 0°C during the storage. Data were represented as mean ± SEM. Statistical significance was assessed by one‐way ANOVA and Tukey's multiple comparison test with *^*^ *p*<0.01 and **^*^ *p*<0.001 when compared with untreated controls

Next, we measured the effects of ultrasonic treatment on the physico‐chemical parameters of whelk meat including pH, WHC, and extent of lipid oxidation. As shown in Figure 5a, the initial pH value of the samples after an ultrasonic treatment was 6.88 ± 0.14 while a similar pH value was recorded in the untreated control samples (6.73 ± 0.13). In the process of storage up to 30 days, both treated and untreated whelk meat samples showed a similar initial sharp decrease in pH due to the formation of lactate and pyruvate by glycolysis. Then, there was an increase in pH after 10 days’ storage time, probably due to the release of basic amino acids during the decomposition of the whelk muscle protein. However, the rise of pH in the control was significantly faster than the ultrasonic‐treated one (Figure 5a), suggesting that the ultrasonic treatment could delay the whelk meat deterioration during storage.

The water‐holding capacity in meat is responsible for its tenderness and juiciness. As shown in Figure 5b, ultrasonic‐treated whelk meat demonstrated significant higher WHC when compared with the untreated samples, while the cooking yield of whelk meat was also increased upon ultrasonic treatment (Figure 5c). These results implied that ultrasonic treatment might also improve water‐holding properties and decrease the cooking loss of whelk meat, similar to that of the ultrasonic‐treated minced beef (*M. semimembranosus*) (Stadnik, Dolatowski, & Baranowska, 2008) and porcine meat (Siró et al., 2009). The results are consistent with the fact that the application of ultrasonic treatment has the potential to increase the water‐holding properties of meat in general (Mcclements, 1995).

2‐Thiobarbituric acid‐reactive substances (TBARS) are the major oxidation products of fatty acids during storage of meat. Therefore, TBARS value can be applied as an indicator to reflect the extent of lipid oxidation in meat. Here, the initial values of TBARS were 0.687 ± 0.055 and 0.623 ± 0.022 mg MDA/kg sample for control and ultrasonic‐treated whelk meat (Figure 5d), respectively. During storage, the TBARS values of all samples had a sharp increase, suggesting that lipid oxidation had occurred during the storage period. However, no significant differences in the TBARS values were observed between treated and untreated samples (Figure 5d), although previous study has suggested that ultrasonic processing might form free radicals which would initiate lipid oxidation during food processing (Chemat, Grondin, Sing Shum Cheong, & Smadja, 2004). However, the present results indicated that the deteriorative effect of lipid oxidation induced by ultrasonic treatment in whelk meat seemed to be not significantly different from the control (Figure 5d).

### 3.5 Microbial analysis of ultrasonic‐treated whelk meats

The total microbial count is one of the important hygiene indicator for meats. Changes in microbial counts on whelk meats with different ultrasonic treatments during storage are shown in Figure 5e. The total microbial count of the two groups showed a clear rising trend over time. There was no significant difference between control and ultrasonic treatment groups on day 0, but total viable count of treatment group was significantly lower than control group after 15 day's of storage. This was consistent with other previous findings in ultrasonic‐treated meats which could delay the growth of microorganisms (Chemat, Zill‐e‐Huma, & Khan, 2011), suggesting the bacteriostatic function of ultrasonic treatment in addition to its tenderization effect on whelk meat.

### 3.6 Sensory evaluation of ultrasonic‐treated whelk meats

After ultrasonic treatment, both untreated control samples and treated samples with lower shear force were submitted to sensory evaluation. As shown in Table 3, the mean sensory scores in terms of hardness, texture, and overall acceptability were significantly higher in ultrasonic‐treated samples, suggesting that they were more acceptable comparing with the untreated controls. Moreover, there were significant differences in the sensory scores for hardness and texture between treated and untreated whelk meat samples (Table 3), suggesting that the lower shear force might contribute to the better sensory evaluation results in the ultrasonic‐treated whelk meat.

**Table 3:** Mean sensory scores of untreated and ultrasonic‐treated whelk meat

	Color	Odor	Hardness	Texture	Overall acceptability
Control	8.96 ± 0.81^a^	9.24 ± 1.20^a^	8.20 ± 2.08^a^	8.32 ± 1.54^a^	8.52 ± 1.26^a^
Ultrasonic‐treated	8.80 ± 1.62^a^	9.16 ± 1.40^a^	9.04 ± 1.22^b^	9.08 ± 1.08^b^	9.16 ± 1.06^b^

Values in the same column with different superscript lower‐case letters differ significantly from each other. Significance was determined using student’s t test (p < 0.05).

## 4 DISCUSSION

Meat tenderness is generally considered as a major determinants of meat quality (Kemp & Parr, 2012) and has the greatest influence on consumer's acceptability. The toughness of whelk meat is one of its undesirable qualities for most consumers. The use of exogenous proteolytic enzymes to enhance tenderness of meat has been conducted for many decades. Currently, only five exogenous proteolytic enzymes isolated from plants (Papain, Bromelain, Ficain) and bacteria (from Bacillus) and fungi (from Aspergillus) have been classified as “Generally Recognized as Safe” (GRAS) by USDA's Food Safety Inspection Service (FSIS) to be used to soften the meat (Ha et al., 2012). However, these enzymatic methods often affect the appearance and quality of the meat (Kemp & Parr, 2012). Therefore, alternative methods such as phosphates (Baker and Darfler, 1968), electrolytes (Lyon and Hamm, 1986), and pressure treatment (Mendiratta and Panda, 1995) have been proposed to improve the meat tenderness. However, many of these treatments have limited practical applications due to their negative effect on the sensory attributes of whelk meat.

Having the characteristics of high efficiency, inexpensive, and environmentally friendly, the use of ultrasonic treatment to improve meat tenderness has attracted much interest recently (Jayasooriya et al., 2004, 2007). In the present study, the RSM approach for modeling and optimizing a response that is affected by one or more factors (Wang et al., 2007; Kim et al., 2013; Hu et al., 2014), has been employed to optimize 3 variables including ultrasonic power, treatment duration and temperature on tenderization of whelk meat. The present results showed that ultrasonic treatment at 200 W and 45°C for 9.6 min led to a significant decrease in the shear force of whelk meat, which was comparable to the commercially used papain and bromelain. These results are in agreement with previous works, showing exposure to ultrasonic treatment is a promising way for meat tenderization. Meanwhile, our results also revealed ultrasonic‐treated whelk meat samples would have a lower pH increase and total microbial count during storage as well as an improved water‐holding properties but no significant increase in lipid oxidation when compared with untreated samples.

Notably, the increase in WHC of ultrasonic‐treated meats was also reported by several independent studies (Hu et al., 2014; Siró et al., 2009; Zhang et al., 2016). Previous studies have also shown that increasing the space between the filaments can up‐regulates the amount of water being retained by the muscle (Yogesh, Jha, & Yadav, 2012). Therefore, more WHC observed in ultrasonic‐treated whelk meat may be at least partially due to the disruption of muscle microstructure and larger cavities in whelk meat as revealed by scanning electron micrographs in our study. In addition, several studies have already shown the relationship between muscle fiber disruption and meat hardness (Bugeon, Lefevre, & Fauconneau, 2003; Hu et al., 2014). For example, high‐frequency ultrasound can lead to physical weakening of the fiber structure during the tenderization of beef (Got et al., 1999). Therefore, this disruption of microstructure as observed may also contribute to the decrease in the shear force of ultrasonic‐treated whelk meat.

## 5 CONCLUSIONS

In conclusion, this study demonstrated a significant decrease in whelk meat hardness as reflected by reduced shear force and an improved overall acceptability were observed in tenderized whelk samples being subjected ultrasonic treatment when compared with the untreated control. The findings in the present study show that ultrasonic treatment is a promising tenderization method for meat with tough muscle fiber like the one found in whelk. However, more investigations are required to understand the detailed changes of the muscle fiber proteins in the whelk meat during the ultrasonic treatment and to apply this technology to the food industry.

## ETHICAL STATEMENT

I testify on behalf of all co‐authors that this article is original and has not been published and will not be submitted for publication elsewhere; research in this article does not involve human experimentation and/or animal testing; all authors have been personally and actively involved in substantive work leading to the manuscript and will hold themselves jointly and individually responsible for its content; all authors have read, approved and are fully conversant with the manuscript; all authors were also aware of its submission to *Food Science & Nutrition* and declared no conflict of interest.

## CONFLICTS OF INTEREST

The authors have declared no conflict of interest.
